# Histomorphological development and progression of gastric intramucosal papillary adenocarcinoma—a retrospective study

**DOI:** 10.3389/fonc.2025.1681155

**Published:** 2025-12-01

**Authors:** Yang-Kun Wang, Xiong-Fei Zou, Wen-Yi Wang, Ying-Ying Li, Yan Zhong, Pei Guo, Tao Wang, Si-Liang Xu, Su-Nan Wang

**Affiliations:** 1 Department of Pathology, The Fourth People’s Hospital of Longgang District, Shenzhen, China; 2 Shenzhen Polytechnic University, Shenzhen, China; 3 Department of Pathology, Southern Medical University Shenzhen Hospital, Shenzhen, China; 4 Department of Pathology, Weifang Army 80th Group Military Hospital, Weifang, China

**Keywords:** immunohistochemistry, gastric neoplasms, gastric-type adenocarcinoma, neoplastic transformation, pathological features

## Abstract

**Objective:**

The aim of this study is to elucidate the developmental process, progression patterns, and histomorphological features of gastric-type intramucosal papillary adenocarcinoma.

**Methods:**

This study employed a retrospective research method. Histological examination and immunohistochemical analyses were conducted on endoscopic biopsy samples and endoscopic submucosal dissection (ESD) specimens from 450 cases of superficial gastric epithelial lesions.

**Results:**

Atrophic changes in gastric foveolar epithelium, resulting from infectious, chemical, autoimmune, or genetic factors, were associated with a sequential process of tumorigenesis in gastric intramucosal papillary adenocarcinoma. This process involved two phases of compensatory epithelial proliferation followed by three distinct stages of neoplastic transformation. The initial compensatory phase was characterized histologically by papillary hyperplasia of the gastric crypt epithelium. The second phase, representing transitional or dysregulated proliferation, was observed as gastric surface epithelial-type adenoma. The first neoplastic stage was identified as gastric-type low-grade intraepithelial neoplasia, followed by high-grade intraepithelial neoplasia, and culminating in the third stage as gastric-type intramucosal papillary adenocarcinoma. The progression of these stages was delineated based on histopathological features and immunophenotypic profiles.

**Conclusion:**

Recognizing the histopathological and immunophenotypic features involved in the stepwise development of gastric-type papillary adenocarcinoma enhances the accuracy of clinical management and surveillance of neoplastic progression. This has significant implications for early intervention and prevention of gastric cancer progression.

## 1 Introduction

Gastric carcinogenesis is a multistep and multifactorial process driven by complex interactions between genetic predisposition and environmental factors. Recognition of predisposing conditions and precursor lesions is critical for effective screening and early intervention strategies. Gastric cancer (GC) remains highly prevalent across Asia and has shown an increasing incidence in the United States (1–3). The limited efficacy of surgical intervention in advanced-stage GC has prompted ongoing efforts to improve early detection and develop more effective therapeutic approaches (4, 5).

Gastric adenocarcinoma (GA), which originates from the gastric mucosal epithelium, comprises multiple histological subtypes. Based on the Lauren classification, intestinal-type GA accounts for approximately 64.6%, diffuse-type for 24.6%, and mixed-type for 10.8% (6, 7). Gastric-type adenocarcinoma, including the foveolar subtype, is relatively uncommon. Foveolar-type adenocarcinoma, particularly in cases managed with endoscopic submucosal dissection (ESD), has been associated with favorable clinical outcomes. This neoplasm is typically diagnosed at an early stage and is characterized by a high degree of differentiation, with malignant epithelial cells exhibiting morphological features resembling normal foveolar or pyloric epithelium. Despite its clinical relevance, there is limited awareness of its histopathological features among pathologists, and few studies have evaluated interobserver variability in its diagnosis (8, 9).

Hyperplasia of the gastric surface (foveolar) epithelium arises in response to a range of etiological factors. Existing clinicopathological literature on foveolar hyperplasia (FH) is largely descriptive and often lacks specificity regarding the extent and severity of mucosal injury. This limitation hinders the development of tailored clinical interventions. Prior investigations have shown that epithelial proliferation in the gastric surface epithelium, triggered by various stimuli, results in distinct and recognizable morphological changes. The etiology and progression patterns of FH have encompassed several subtypes, including ordinary FH, drug-induced FH, *Helicobacter pylori* (HP)-associated FH, metaplastic FH, and atrophic FH. In addition, cases involving foveolar epithelial neoplasia and proliferative transformation have also been documented, along with progression to signet-ring cell carcinoma originating from the foveolar epithelium (10–12).

This study explored the formation and underlying mechanisms of early-stage gastric-type adenocarcinoma using histomorphological assessment and immunohistochemical analysis. The resulting findings offer pathological insights relevant to guiding individualized therapeutic strategies and facilitating the surveillance of proliferative transformation, thereby contributing to the control of foveolar epithelial carcinoma development and progression.

## 2 Materials and methods

### 2.1 Clinical data

This study employed a retrospective research method. A total of 450 patients diagnosed with gastric surface epithelial lesions by histopathological evaluation were included in this study. Specimens were collected between December 2023 and February 2025 from Shenzhen Longgang District Fourth People’s Hospital, Shenzhen Hospital of Southern Medical University, and the 80th Army Group Hospital. This study was conducted with approval from the Ethics Committee of The Fourth People’s Hospital of Longgang District (Approval No. LGKCYLWS2023034).

Inclusion criteria: (1) Age between 21 and 76 years old; (2) Patients do not have other malignant tumors; (3) Patients have no history of gastric ulcers or other diseases; (4) Patients or their family members have signed informed consent; (5) Basic information of patients is complete.

Exclusion criteria: (1) Patients are pregnant; (2) Patients’ information is incomplete.

Among these cases, 9 involved gastric-type high-grade intraepithelial neoplasia, 2 were diagnosed as gastric-type intramucosal papillary adenocarcinoma, and 6 presented with mixed gastric intestinal-type intramucosal papillary adenocarcinoma; all 17 underwent ESD. The remaining 433 cases did not reach the level of high-grade intraepithelial neoplasia. All pathological results are independently diagnosed by two pathologists with intermediate or higher professional titles. In case of any disagreement, the department head has the final decision-making power.

Three to five mucosal tissue specimens were biopsied from each site. Specimens were fixed in 10% neutral buffered formalin, processed routinely, embedded in paraffin, sectioned at 4 μm thickness, and stained with hematoxylin-eosin (H&E) and underwent immunohistochemical analysis.

### 2.2 Histomorphological staging

Histopathological diagnoses were established in accordance with the 2019 *World Health Organization Classification of Digestive System Tumours* and recognized criteria for gastric tumor pathology (13, 14). Lesions were classified based on the developmental sequence and progression of foveolar hyperplastic-to-neoplastic transformation, as well as the degree of dysplastic epithelial proliferation. Lesions were classified into seven sequential histological stages reflecting the progression from precursor lesions to invasive carcinoma: (1) foveolar epithelial atrophy, (2) foveolar papillary hyperplasia, (3) foveolar polyp/surface epithelial adenoma, (4) foveolar low-grade intraepithelial neoplasia, (5) foveolar high-grade intraepithelial neoplasia, (6) foveolar intramucosal papillary adenocarcinoma, and (7) mixed gastric-intestinal type intramucosal papillary adenocarcinoma.

### 2.3 Immunohistochemical staining

To distinguish between gastric and intestinal epithelia, immunohistochemical methods are employed. Immunohistochemical staining was conducted using the EnVision two-step method. Primary antibodies included those against HP, CK7, CK20, CEA, MUC5AC, MUC1, MUC2, MUC6, Villin, CDX2, p53, and Ki-67. All reagents and antibody solutions were purchased from Guangzhou Xiuwei Technology Co., Ltd. Staining procedures were carried out in strict accordance with the manufacturer’s instructions.

## 3 Results

### 3.1 Clinical features

Among the 450 patients included in the study, 278 were male and 172 were female. Lesion locations included the gastric angle (31 cases), antrum (24 cases), body (17 cases), and fundus (11 cases). Patients aged ≤ 60 years comprised 55.8% (251/450) of the cohort, while those aged > 60 years accounted for 44.2% (199/450) (**Table 1**).

**Table 1:** Staging of gastric intramucosal papillary adenocarcinoma: distribution by sex and age.

Stage	N (%)	Gender		Age Group	
		Male	Female	≤60	>60
Foveolar epithelial atrophy	182(44.8)	113(62.1)	69(37.9)	105(57.7)	77(42.3)
Papillary hyperplasia of gastric surface epithelium	174(19.4)	106(60.9)	68(39.1)	98(56.3)	76(43.7)
Gastric surface epithelial adenoma	35(8.3)	22(62.9)	13(37.1)	20(57.1)	15(42.9)
Gastric-type low-grade intraepithelial neoplasia	42(1.3)	26(61.9)	16(38.1)	22(52.4)	20(47.6)
Gastric-type high-grade intraepithelial neoplasia	9(1.3)	6(66.7)	3(33.3)	4(44.4)	5(55.6)
Gastric-type intramucosal papillary adenocarcinoma	2(1.3)	1(50.0)	1(50.0)	0	2(100)
Mixed gastric-intestinal type intramucosal papillary adenocarcinoma	6(1.3)	4(66.6)	2(33.3)	2(33.3)	4(66.6)
Total	450	278(61.8)	172(38.2)	251(55.8)	199(44.2)

### 3.2 Development and progression of gastric-type papillary carcinoma

**Figure 1:**
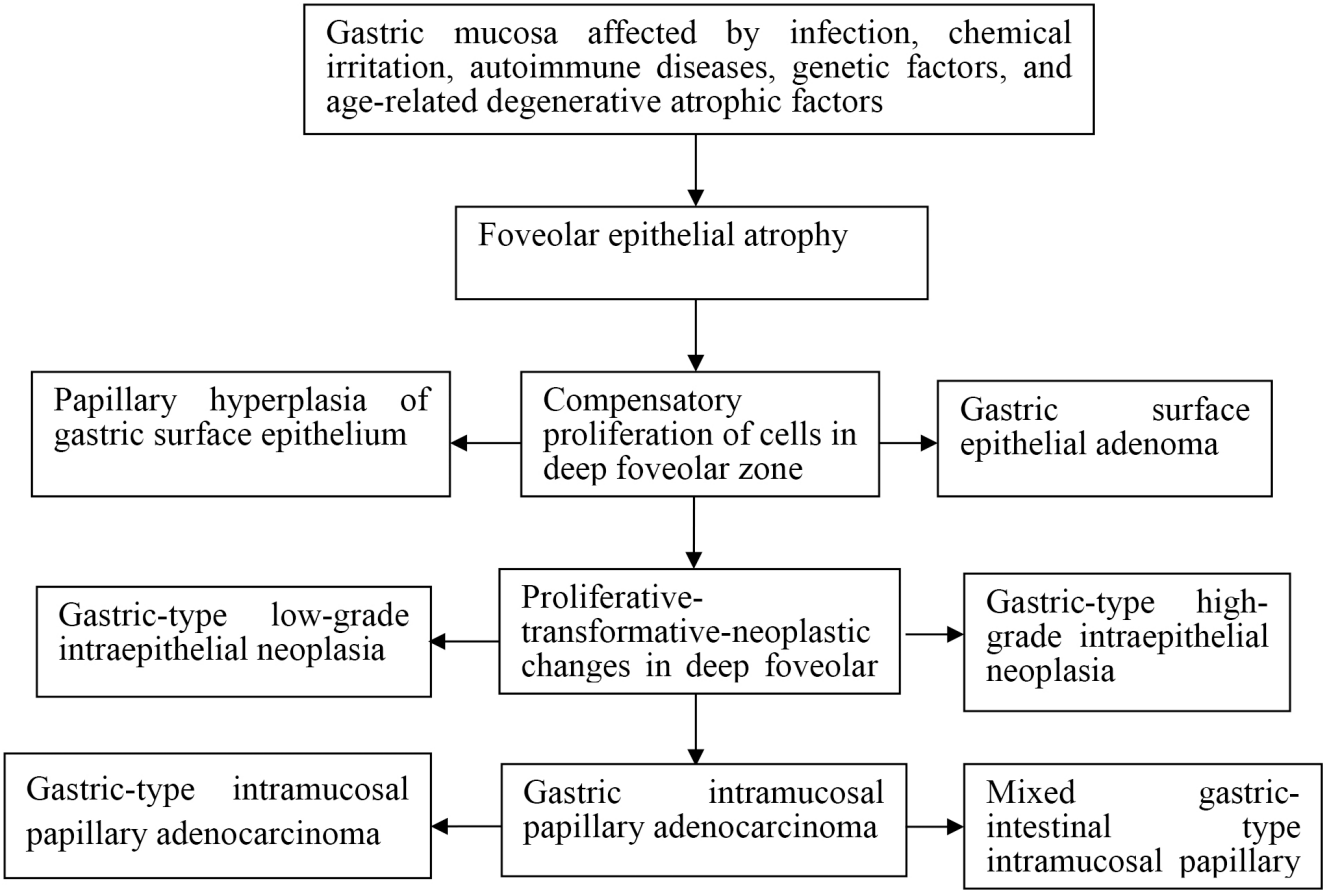
Schematic diagram of development and progression of gastric intramucosal papillary adenocarcinoma and its histomorphological characteristics.

Exposure of the gastric mucosa to infectious agents, chemical irritants, autoimmune responses, or inherited genetic abnormalities was associated with the development of foveolar epithelial atrophy. This atrophic change was followed by compensatory proliferation within the deep foveolar zone, which progressed through stages of proliferative transformation and neoplastic progression, ultimately resulting in the formation of intramucosal papillary adenocarcinoma (**Figure 1**).

### 3.3 Histomorphological features of gastric-type papillary carcinoma development

Seven distinct histological stages were delineated in the progression from foveolar epithelial atrophy to intramucosal papillary adenocarcinoma.

**Figure 2:**
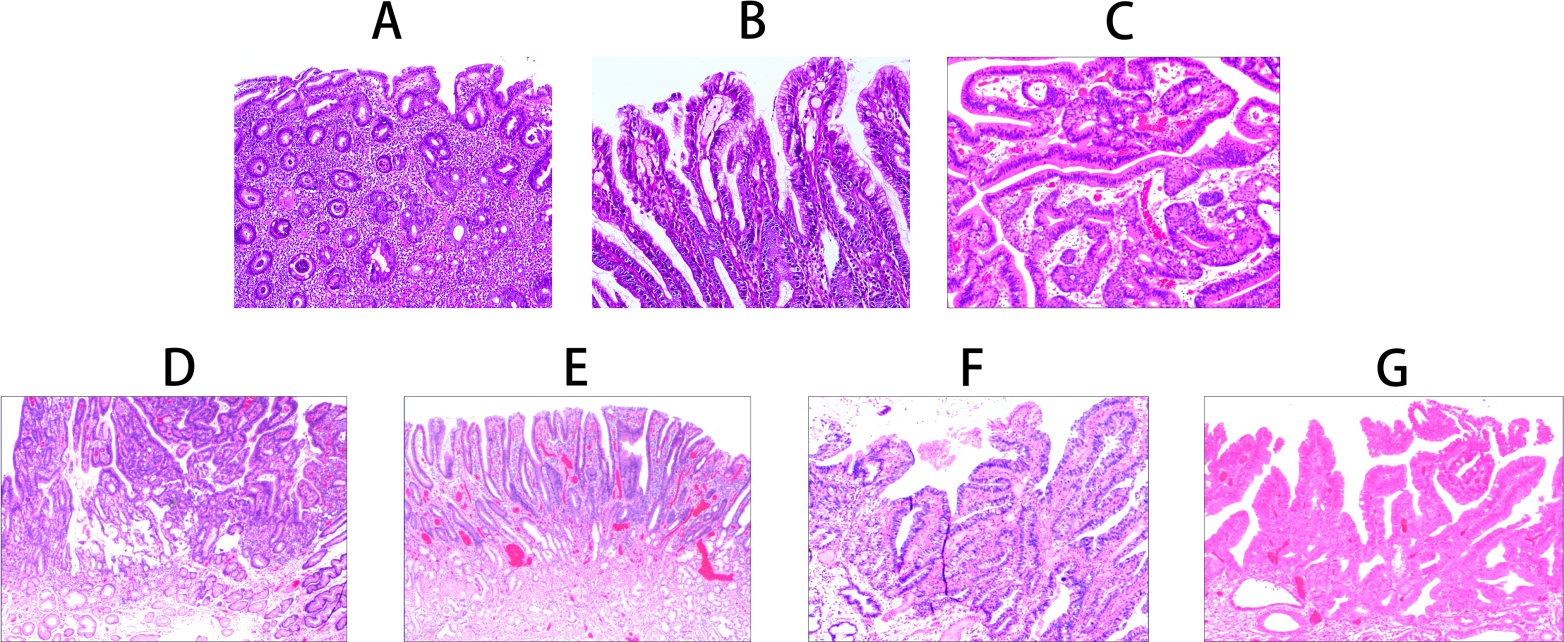
Histomorphological characteristics of gastric-type papillary carcinoma development and progression. **(A)** Foveolar epithelial atrophy: Atrophy of the foveolar epithelial layer, complete loss in severe cases (H&E staining ×100). **(B)** Papillary hyperplasia of gastric surface epithelium: Mixed proliferation of foveolar and crypt epithelium forming well-polarized papillary structures (H&E staining ×200). **(C)** Gastric surface epithelial adenoma: Mixed proliferation of foveolar and crypt epithelium forming disorganized papillary structures (H&E staining ×200). **(D)** Gastric-type low-grade intraepithelial neoplasia: Glandular epithelial cells irregularly arranged with enlarged nuclei and increased nuclear-cytoplasmic ratio (H&E staining ×100). **(E)** Gastric-type high-grade intraepithelial neoplasia: Proliferative cells with enlarged nuclei and increased nuclear-cytoplasmic ratio, with 30-50% showing enlarged prominent nucleoli (H&E staining ×100). **(F)** Gastric-type intramucosal papillary adenocarcinoma: Proliferative transformed glandular epithelium with morphological characteristics of both foveolar and crypt epithelium (H&E staining ×100). **(G)** Mixed gastric-intestinal type intramucosal papillary adenocarcinoma: Proliferative transformed glandular epithelium with mixed morphological characteristics of foveolar, crypt, and intestinal epithelium (H&E staining ×100).

Foveolar epithelial atrophy is histologically characterized by the thinning of the foveolar epithelial layer. In advanced cases, there is discontinuity of the epithelial layer (note: this change is only observed in severe cases with long-term etiological exposure, confirmed by double-blind review and quality control procedures to rule out artificial interference) (**Figure 2A**). Papillary hyperplasia of the gastric surface epithelium shows mixed proliferation of foveolar and crypt epithelium, forming well-polarized papillary structures (**Figure 2B**). Gastric surface epithelial adenoma demonstrates mixed proliferation of foveolar and crypt epithelium, forming disorganized papillary structures (**Figure 2C**).

In gastric-type low-grade intraepithelial neoplasia, epithelial cells show cytological and architectural atypia with nuclear enlargement, an increased nuclear-to-cytoplasmic ratio, and prominent nucleoli (**Figure 2D**). Gastric-type high-grade intraepithelial neoplasia is characterized by proliferative cells with nuclear enlargement and an increased nuclear-cytoplasmic ratio. Approximately 30%–50% of cells display prominent nucleoli, while 5%–20% contain intranuclear eosinophilic inclusions. Mitotic figures are observed at a frequency of 2–3 per high-power field (HPF), and the Ki-67 labeling index ranged from 30%–60% (**Figure 2E**).

Gastric-type intramucosal papillary adenocarcinoma exhibits neoplastic glandular epithelium with morphological and immunophenotypic features of both foveolar and crypt epithelium. The nuclei are basally located, irregular in shape, and crowded, with 1–2 nucleoli and mitotic figures at a frequency of 3–6/HPF (**Figure 2F**). The mixed gastric-intestinal type intramucosal papillary adenocarcinoma shows neoplastic glandular epithelium with combined morphological and immunophenotypic features of foveolar, crypt, and intestinal epithelium (**Figure 2G**) (**Table 2**).

**Table 2:** Staging and histopathological characteristics of gastric intramucosal papillary adenocarcinoma.

Lesion type	Histopathological type	Histopathological characteristics
Foveolar epithelial atrophy	–	Characterized by destruction and loss of the papillary structure of the foveolar epithelium, typically resulting from chronic infection or chemical irritation to the gastric mucosa. Histological examination reveals a marked reduction in foveolar epithelial cells. Normal architecture can be restored after elimination of causative factors.
Compensatory proliferation of cells in the deep foveolar zone	–	Persistent mucosal injury resulting from infection or chemical irritation promoted compensatory epithelial cell proliferation in the deep foveolar zone, as well as in the isthmus and neck regions of the gastric glands. Morphologically, this manifests mainly as compensatory proliferation and dysregulated compensatory proliferation.
	Papillary hyperplasia of the gastric surface epithelium	Enhanced upward migration of cells from the proliferative regions of the deep foveolar zone, gastric gland isthmus, and neck, forming compensatory hyperplasia. Hyperplastic glands and cells maintain normal polarity, also termed polar compensatory proliferation. Histologically, mixed proliferation of foveolar and crypt epithelium forms well-polarized papillary structures. Normal architecture can be restored after removal of causative factors.
	Gastric surface epithelial adenoma	Enhanced upward migration of cells from proliferative regions of the deep foveolar zone, gastric gland isthmus, and neck, forming persistent compensatory hyperplasia. Hyperplastic glands and cells show transitional compensatory proliferation and/or dysregulated compensatory proliferation. Histologically, mixed proliferation of foveolar and crypt epithelium forms disorganized papillary structures, also termed neoplastic proliferation. Regression was possible following resolution of the injurious stimuli.
Proliferative-transformative-neoplastic changes in the deep foveolar zone cells	–	Continued exposure to pathogenic or chemical factors induced proliferative transformation of stem cells within the deep foveolar zone, gastric gland isthmus, and neck. Morphologically, this manifests mainly as gastric-type low-grade intraepithelial neoplasia and gastric-type high-grade intraepithelial neoplasia.
	Gastric-type low-grade intraepithelial neoplasia	First proliferative transformation of stem cells in proliferative regions of the deep foveolar zone, gastric gland isthmus, and neck, showing varying degrees of cytological and architectural atypia. Histological features included irregular glandular arrangement, nuclear enlargement, an increased nuclear-to-cytoplasmic ratio, and prominent enlarged nucleoli.
	Gastric-type high-grade intraepithelial neoplasia	Gastric-type high-grade intraepithelial neoplasia refers to further development of stem cells in proliferative regions, representing the second proliferative transformation process. This transformation is characterized mainly by mixed continuation of foveolar and crypt epithelium. Abnormally proliferative transformed cells form irregular glandular or branching structures. Histologically, nuclei are enlarged with increased nuclear-cytoplasmic ratio; approximately 30–50% show enlarged prominent nucleoli; 5–20% contain intranuclear eosinophilic inclusions. Mitotic figures: 2-3/HPF; Ki-67 positive cells: 30–60%. This lesion demonstrates irreversible proliferation, and ESD resection is recommended.
Intramucosal papillary adenocarcinoma	–	When gastric mucosa is persistently affected by infection and chemical irritation, stem cells in proliferative regions of the deep foveolar zone, gastric gland isthmus, and neck undergo a third proliferative transformation process. Since proliferative zones contain various poorly differentiated cells, morphologically this manifests mainly as gastric-type intramucosal papillary adenocarcinoma and mixed gastric-intestinal type intramucosal papillary adenocarcinoma.
	Gastric-type intramucosal papillary adenocarcinoma	Gastric-type intramucosal papillary adenocarcinoma refers to continued development of stem cells in proliferative regions, mainly involving mixed proliferative transformation of foveolar and crypt epithelium. Histologically, proliferative transformed glandular epithelium exhibits morphological and immunophenotypic features characteristic of both foveolar and crypt epithelium. Nuclei are basally located; in actively proliferating areas, appear hyperchromatic, irregular in shape, and densely crowded, with 1–2 visible nucleoli and 3–6 mitotic figures per HPF.
	Mixed gastric-intestinal type intramucosal papillary adenocarcinoma	Mixed gastric-intestinal type intramucosal papillary adenocarcinoma refers to continued development of stem cells in proliferative regions, mainly involving mixed proliferative transformation of foveolar, crypt, and intestinal epithelium. Histologically, proliferative transformed glandular epithelium displays mixed morphological characteristics and immunophenotype of foveolar, crypt, and intestinal epithelium. Nuclei are basally located, irregular in shape, and hyperchromatic, with 1–2 nucleoli and 3–6 mitotic figures per HPF.

### 3.4 Immunohistochemical results

Immunohistochemical evaluation demonstrated stage-specific expression patterns of diagnostic markers throughout the progression of gastric-type lesions.

**Figure 3:**
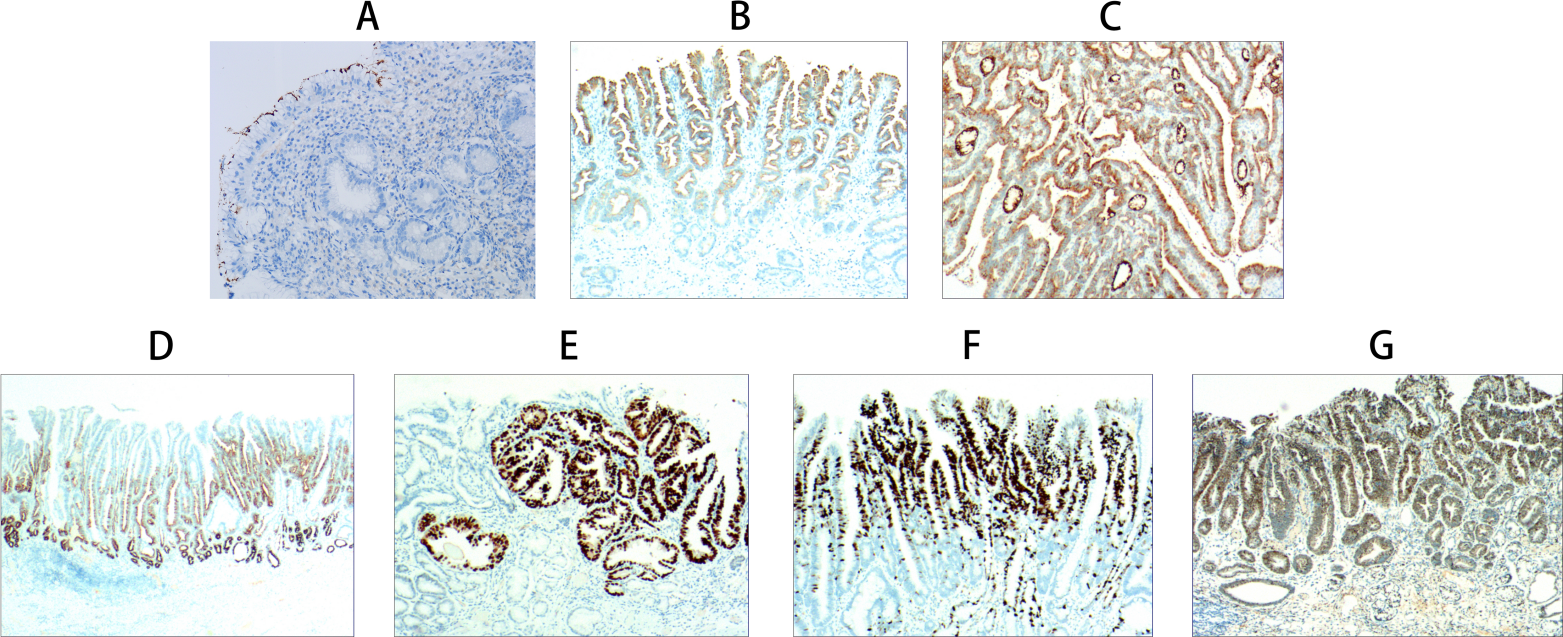
Immunohistochemical staining results of gastric-type papillary carcinoma development and progression. **(A)** Foveolar epithelial atrophy: Atrophy of foveolar epithelial layer with only one epithelial layer remaining and loss of foveolar. HP positive expression (EnVision method, ×200, ×100). **(B)** Papillary hyperplasia of gastric surface epithelium: MUC1 positive expression (EnVision method, ×40). **(C)** Gastric surface epithelial adenoma: MUC5AC positive expression (EnVision method, ×100). **(D)** Gastric-type low-grade intraepithelial neoplasia: MUC6 positive expression (EnVision method, ×20). **(E)** Gastric-type high-grade intraepithelial neoplasia: p53 positive expression (EnVision method, ×100). **(F)** Gastric-type intramucosal papillary adenocarcinoma: Ki-67 positive cells 60-70% (EnVision method, ×100). **(G)** Mixed gastric-intestinal type intramucosal papillary adenocarcinoma: CDX2 positive expression (EnVision method, ×100).

In foveolar epithelial atrophy, positive staining for HP confirmed the presence of HP infection (**Figure 3A**). Papillary hyperplasia of the gastric surface epithelium exhibited positive MUC1 expression (**Figure 3B**), while gastric surface epithelial adenoma demonstrated positivity for MUC5AC (**Figure 3C**).

Gastric-type low-grade intraepithelial neoplasia showed co-expression of carcinoembryonic antigen (CEA) and MUC6 (**Figure 3D**). In gastric-type high-grade intraepithelial neoplasia, p53 positivity was observed (**Figure 3E**). Gastric-type intramucosal papillary adenocarcinoma was associated with a Ki-67 labeling index ranging from 60%–70%, indicating high proliferative activity (**Figure 3F**).

The mixed gastric-intestinal type intramucosal papillary adenocarcinoma displayed immunopositivity for MUC2, villin, and CDX2, consistent with features of intestinal differentiation. (**Figure 3G**) (**Table 3**).

**Table 3:** Immunohistochemical profiles of gastric intramucosal papillary adenocarcinoma stages.

Pathological stage	HP	CEA	MUC5AC	MUC1	MUC2	MUC6	Villin	CDX2	p53	ki67
Foveolar epithelial atrophy	-/+	–	+	+	–	–	–	–	–	–
Papillary hyperplasia of gastric surface epithelium	-/+	–	+	+	–	–	–	–	–	–
Gastric surface epithelial adenoma	-/+	-/+	+	+	–	–	–	–	–	0~2%
Gastric-type low-grade intraepithelial neoplasia	-/+	+	+	+	–	+	–	–	–	5~10%
Gastric-type high-grade intraepithelial neoplasia	-/+	+	+	+	–	+	–	–	-/+	15~40%
Gastric-type intramucosal papillary adenocarcinoma	-/+	+	+	+	–	+	–	–	+	30~60%
Mixed gastric-intestinal type intramucosal papillary adenocarcinoma	-/+	+	-/+	-/+	-/+	+	+	+	+	40~80%

## 4 Discussion

The fifth edition of the *World Health Organization Classification of Digestive System Tumours* (2019) introduced gastric foveolar (crypt) dysplasia within the section on benign epithelial tumors and precancerous lesions of the stomach (13). Gastric mucosal epithelial dysplasia manifests as four types: adenomatous, crypt, regenerative, and cystic. Among these, gastric crypt dysplasia represents a distinct pathological entity (15). Previous studies have indicated a frequent association between gastric-type adenocarcinoma and crypt epithelial dysplasia, suggesting that crypt dysplasia serves as both a key precursor lesion and a histomorphological intermediary in the gastric carcinogenic pathway (16–18).

GC typically arises from a continuum of precursor lesions such as mucosal atrophy, intestinal metaplasia, epithelial dysplasia, and chronic inflammatory cell infiltration, each contributing to the emergence of diverse histopathological patterns (19–22). However, comprehensive investigations detailing the carcinogenic sequence and histological progression of lesions originating from the gastric crypt epithelium remain limited.

In the present study, exposure of the gastric mucosa to infectious or chemical irritation, autoimmune disease, or genetic factors was found to induce foveolar epithelial hyperplasia. In cases involving sustained or severe injury, progression to foveolar epithelial atrophy occurred. This was followed by compensatory proliferation of epithelial cells within the deep foveolar zone, which histologically manifested as papillary hyperplasia and neoplastic proliferation of the crypt epithelium.

The developmental trajectory of gastric intramucosal papillary adenocarcinoma (GIPA) appeared to proceed through four consecutive stages: (1) foveolar epithelial atrophy; (2) compensatory proliferation of deep foveolar zone cells; (3) proliferative, transformative, and neoplastic changes within the deep foveolar epithelium; and (4) the emergence of intramucosal papillary adenocarcinoma.

Seven distinct histomorphological patterns were observed during the progression from foveolar epithelial atrophy to intramucosal papillary adenocarcinoma: (1) Foveolar epithelial atrophy: characterized by thinning or complete loss of the foveolar epithelial layer. Proliferative activity was absent or markedly reduced in the deep foveolar zone, gastric gland isthmus, and neck regions, although the overall glandular architecture remained largely preserved. (2) Papillary hyperplasia of crypt epithelium: marked by enhanced upward migration of epithelial cells from the deep foveolar zone, isthmus, and neck proliferative regions. This resulted in compensatory hyperplasia with preserved glandular polarity, referred to as polar compensatory hyperplasia. (3) Crypt epithelial adenoma: represented a continuation of the compensatory hyperplasia, with sustained upward migration and proliferation of epithelial cells. Glandular and cellular architecture demonstrated transitional or dysregulated features, consistent with neoplastic proliferation. (4) Gastric-type low-grade intraepithelial neoplasia: in this stage, epithelial cells in the deep foveolar zone cells exhibited proliferative, transformative, and neoplastic changes, accompanied by varying degrees of cytological and architectural atypia. (5) Gastric-type high-grade intraepithelial neoplasia: further progression of proliferative-transformative-neoplastic changes in the deep foveolar zone cells. (6) Gastric-type intramucosal papillary adenocarcinoma: developed through excessive proliferation of mucous neck cells located in the gastric gland isthmus and neck regions. (7) Mixed gastric-intestinal type intramucosal papillary adenocarcinoma: arising from aberrant proliferation of stem cells within gastric proliferative zones.

Gastric intramucosal adenocarcinoma represents an early stage of GC and is commonly treated with ESD (23, 24). Papillary adenocarcinoma is characterized by distinct papillary or villous architecture. Previous studies have demonstrated that papillary GA is associated with specific clinical and molecular characteristics, including older age at diagnosis, elevated carcinoembryonic antigen levels, differentiation grade, perineural invasion, overexpression of human epidermal growth factor receptor 2 *(HER2)*, and *P53* gene mutation status (25).

A documented case of early-stage gastric papillary adenocarcinoma demonstrated disease recurrence following curative surgical resection. Although histopathological assessment revealed intramucosal cancer without evidence of lymphovascular invasion or lymph nodal metastasis, imaging performed 8 months postoperatively revealed osteolytic lesions in the right sacrum, and biopsy confirmed bone metastasis (26).

Early GA often lacks the cellular morphology, histological architecture, and tumor microenvironment components including fibroblasts, macrophages, cytokines, chemokines, and T lymphocytes that are typically observed in advanced malignancy, contributing to substantial variability in diagnostic interpretation (27–29).

Gastric intestinal metaplasia, frequently resulting from chronic inflammation, is recognized as a risk factor for gastric carcinogenesis. Although both HP infection and autoimmune gastritis are established causes of intestinal metaplasia, the metaplastic epithelial phenotypes differ by etiology and exhibit different histopathological features (30–32). HP infection has also been implicated in the development of FH and foveolar adenoma. The progression from intestinal metaplasia to epithelial dysplasia is associated with an increased risk of intestinal-type GA; however, the molecular mechanisms underlying this metaplastic-to-neoplastic transformation remain incompletely understood (33–35). At present, no published studies have described the formation and pathogenesis of gastric intramucosal papillary adenocarcinoma (GIPA).

This study evaluated gastric mucosal biopsy samples and ESD specimens to investigate the development and progression of intramucosal papillary adenocarcinoma. Severe infectious or chemical injury to the gastric mucosa was associated with disruption of the papillary architecture of the foveolar epithelium. This trigger enhanced upward migration of epithelial cells originating from the deep foveolar zone, glandular isthmus, and mucous neck cells, initially giving rise to compensatory hyperplasia. Morphologically, this phase corresponded to papillary hyperplasia of the crypt epithelium and is referred to as polar compensatory proliferation.

With persistent exposure to injurious stimuli, compensatory hyperplasia progressed to neoplastic proliferation, morphologically manifesting as crypt epithelial adenoma. This stage was characterized by transitional or dysregulated compensatory proliferation. Continued proliferative transformation of gastric stem cells in response to sustained stimulation resulted in the development of gastric-type intraepithelial neoplasia.

Sequential transformation of stem cells within the gastric proliferative zones occurred under prolonged infection and chemical irritation. The first stage of this transformation yielded gastric-type low-grade intraepithelial neoplasia; the second stage resulted in high-grade intraepithelial neoplasia; and the third stage was associated with persistent injury affecting the same proliferative compartments. As these zones contain various poorly differentiated epithelial cell types, the resultant neoplastic architecture displayed features that were consistent with either gastric-type or mixed gastric intestinal-type intramucosal papillary adenocarcinoma.

Gastric-type intramucosal papillary adenocarcinoma develops through sustained proliferative activity of stem cells within gastric proliferative zones, characterized by mixed proliferative transformation involving both foveolar and crypt epithelium. In contrast, mixed gastric-intestinal type intramucosal papillary adenocarcinoma results from combined proliferative transformation of foveolar, crypt, and intestinal epithelial components.

This study has several limitations. First, it was a retrospective analysis based on data from a limited number of centers, which may introduce selection bias. Second, the proposed sequence of histological progression was inferred primarily from cross-sectional observations and histopathological correlations, rather than direct longitudinal follow-up of individual lesions. Third, not all cases progressed to high-grade intraepithelial neoplasia or invasive carcinoma, limiting the assessment of earlier premalignant changes. Finally, due to the absence of an external validation cohort and the lack of a control group, the general applicability of our research results is limited.

## 5 Conclusion

Our findings elucidate a stepwise histomorphological progression from gastric foveolar atrophy to intramucosal papillary adenocarcinoma, identifying key morphological and immunophenotypic stages that can aid in the early detection and risk stratification of gastric neoplasia. Recognition of these stages may inform decisions regarding the timing of endoscopic intervention, such as ESD, particularly in lesions showing high-grade intraepithelial neoplasia or distinct papillary architecture. Furthermore, the identified immunohistochemical markers offer auxiliary tools for precise diagnosis, thereby improving strategies for gastric cancer prevention and control. While ESD is effective for early-stage lesions, our work underscores the importance of surveillance in precursor stages to prevent progression.

Although ESD is a sufficient and curative treatment for early-stage gastric-type papillary adenocarcinoma, our findings provide a histopathological framework that guides the selection and timing of ESD, particularly in premalignant or borderline lesions.

## Data Availability

The original contributions presented in the study are included in the article/Supplementary Material. Further inquiries can be directed to the corresponding authors.
